# Elevated CXCL12 in the plasma membrane of locally advanced rectal cancer after neoadjuvant chemoradiotherapy: a potential prognostic marker

**DOI:** 10.7150/jca.64082

**Published:** 2022-01-01

**Authors:** Sup Kim, Min-Kyung Yeo, Jun-Sang Kim, Ji-Yeon Kim, Kyung-Hee Kim

**Affiliations:** 1 Department of Radiation Oncology, Chungnam National University School of Medicine, 288 Munhwa Street, Daejeon 35015, Korea.; 2 Department of Radiation Oncology, Chungnam National University Hospital, 282 Munwha-ro, Daejeon 35015, Korea.; 3 Department of Pathology, Chungnam National University School of Medicine, 266 Munhwa Street, Daejeon 35015, Korea.; 4 Department of Pathology, Chungnam National University Hospital, 282 Munwha-ro, Daejeon 35015, Korea.; 5 Department of Surgery, Division of Colorectal Surgery, Chungnam National University School of Medicine, Daejeon, Republic of Korea.; 6 Department of Pathology, Chungnam National University Sejong Hospital, 20 Bodeum 7-ro, Sejong-si 30099, Korea.

**Keywords:** CXCL12, plasma membrane, locally advanced rectal adenocarcinoma, neoadjuvant chemoradiotherapy, recurrence

## Abstract

**Background:** Neoadjuvant chemoradiotherapy (nCRT) in locally advanced rectal cancer (LARC) has been shown to improve sphincter preservation and local pelvic control, but the efficacy of nCRT plateaus due to metastasis. CXC chemokine ligand 12 (CXCL12) has a critical impact on cancer development and metastasis.

**Methods:** By investigating public databases containing LARC patient data, CXCL12, CXCR4 and FAPα expression was analyzed via the Tumor Immune Estimation Resource (TIMER) and GSEA. Immunohistochemistry was applied to a total of 121 surgically resected specimens consisting of 61 LARCs after nCRT and 60 LARCs with no nCRT and 16 cases with endoscopic resection of high-grade colorectal adenoma.

**Results:** By investigating public databases containing LARC patient data, CXCL12 expression is correlated with poor prognosis, immune cell infiltration, epithelial- mesenchymal transition, and angiogenesis in LARC. Furthermore, radiation selectively induced CXCL12, CXCR4 and FAPα expression in tumor tissues. Immunohistochemistry results showed that the levels of CXCL12, CXCR4, and FAPα in LARC cells after nCRT were higher than in LARC cells untreated with nCRT (*p* < 0.001 for each). Elevated levels of CXCL12 in the plasma membrane of LARC cells after nCRT demonstrated an association with the period of freedom from recurrence (FFR) in univariate and multivariate survival analyses (*p* = 0.005 and *p* = 0.031, respectively).

**Conclusions:** The expression of CXCL12 may influence the survival and invasive properties of LARC cells during nCRT and promote cancer recurrence. We suggest that CXCL12 expression in the plasma membrane of radioresistant LARC cells may be a predictive factor of recurrence and a viable therapeutic strategy to control radioresistant LARC recurrence.

## Introduction

The current standard treatment for locally advanced rectal cancer (LARC), defined as a stage II (T3-4, node negative) or stage III (node positive) disease, is a multimodality strategy incorporating neoadjuvant chemoradiotherapy (nCRT) followed by total mesorectal excision 1. Although nCRT has been shown to improve sphincter preservation, local pelvic control, and downstaging, the efficacy of this strategy plateaus due to frequent distant metastasis for patients with LARC 2. The initial 9 based staging is one of the important factors for predicting the prognosis of rectal cancer patients. However, this stage less clearly distinguishes groups of patients with different prognoses, particularly for patients in stages II and III of LARC who receive nCRT treatment 3. To overcome the issue of inaccurate prediction, various biological markers have been studied for their ability to forecast responses to preoperative treatment 4. Tumor regression grading, referring to a classification of cancer response to nCRT, has been reported to have a prognostic factor. Furthermore, it was reported that the prognostic value of tumor regression grading may even exceed that of currently used TNM staging, a proposal which originated from analyzing data of untreated tumors 5.

For these reasons, there is growing interest in tumors and tumor microenvironments after nCRT treatment. Accumulating evidence suggests that recruited tumor-associated macrophages promote the recovery of blood flow in irradiated tumors and promote the recurrence of tumors. Recently, it was reported that a critical mechanism for the influx of bone marrow-derived macrophages is driven by the CXC chemokine ligand 12/CXC chemokine receptor type 4 (CXCL12/CXCR4) chemokine pathway 6. While studying CXCL12 expression in colorectal cancer, we found that the increased expression of CXCL12 was in the plasma membrane of the invasive front of residual LARC cells after nCRT in our preliminary examples. We expected CXCL12 to play a special role in the expression of the plasma membrane of LARC cells. CXCL12 and its receptor CXCR4 have been implicated in tumor growth and metastasis in a variety of cancers, and CXCL12 expression has been observed in the tumor microenvironment and cancer cell cytoplasm and plasma membranes, but not focused on the plasma membrane 7-10. We focused on investigating the plasma membrane expression of CXCL12 in LARC cells.

Various studies reported that CXCL12 can be regulated by a number of factors such as HIF-1α, G-CSF, SMAD, and fibroblast activation protein-α (FAPα) 11, 12. A previous study reported that one of these factors is responsible for CXCL12 induction, and there is growing interest in FAPα protein as a potential target for CXCL12-expressing tumors because FAPα in carcinoma-associated fibroblast (CAF) directly controls CXCL12 levels in tumors, and the depletion of CAF-expressing FAPα from tumors permitted immune control of tumor growth in human pancreatic ductal adenocarcinoma 11.

We hypothesized that the expression profiles of CXCL12, CXCR4, and FAPα could provide valuable information regarding cancer invasion in LARC and cancer cell survival related to nCRT resistance in LARC. Hence, this study aimed to investigate CXCL12, CXCR4, and FAPα and their value as cancer survival factors in LARC after nCRT. Therefore, we first evaluated the association of CXCL12, CXCR4, and FAPα mRNA expression with immune cell infiltration and procancer pathways in LARC using the Tumor IMmune Estimation Resource (TIMER) database and GSEA. Then, we checked for changes in CXCL12, CXCR4, and FAPα mRNA expression between pre-nCRT and post-nCRT LARC and adjacent normal tissues using GEO datasets. Finally, we performed immunohistochemical staining for CXCL12, CXCR4, and FAPα in LARC after nCRT and LARC untreated with nCRT and analyzed various clinicopathological characteristics.

## Materials and Methods

### Patients and Tissue Samples

This study was approved by the Institutional Review Board of Chungnam National University Hospital (CNUH 2019-10-041-002). Formalin-fixed paraffin-embedded (FFPE) tissue samples were used for immunohistochemical staining. The biospecimens and data used for this study were provided by the Biobank of Chungnam National University Hospital, a member of the South Korea Biobank Network. The requirement for informed consent for the retrospective comparison study was waived because the study was based on immunohistochemical staining using FFPE tissue. We used representative FFPE whole-tissue samples of 61 LARCs after nCRT and 60 LARCs untreated with nCRT, and 16 cases of high-grade dysplastic colorectal adenoma for immunohistochemical staining. The 61 patients with LARC were treated with preoperative radiotherapy and concurrent capecitabine, followed by mesorectal excision. Of the 61 LARCs treated with nCRT, 45 cases are paired with pre-nCRT biopsy tissue samples. The 61 LARCs after nCRT were in the mid or low rectum and the 60 LARCs untreated with nCRT were in the upper rectum. All 121 cases of LARC were moderately differentiated adenocarcinoma and negative for circumferential resection margin involvement.

The inclusion criteria were that the FFPE tumor tissues were isolated from LARC patients who underwent surgical operation for rectal adenocarcinoma or endoscopic submucosal dissection for colorectal adenoma and that the follow-up clinical data were sufficiently detailed. The exclusion criteria were as follows: (1) patients had a previous history of other cancers; (2) patients had received previous curative resection for any colorectal tumor lesion; (3) patients had received any molecular targeted therapy; (4) surgical curative resection of LARC was positive for circumferential resection margin involvement or pathologic tumor stage 4 (pT4); (5) patients have distant metastasis in the initial diagnosis; or (6) patients have achieved complete pathological response after nCRT.

The pathologic tumor, node, and metastasis (pTNM) staging, histologic grading for LARC and modified Ryan scheme for tumor regression score (R score) were determined at the time of surgical resection and were based on the 8th edition of the American Joint Committee on Cancer (AJCC) staging system 13. The period of freedom from recurrence (FFR) was defined as the interval between the date of surgical resection and the date of the first recurrence or the last follow-up. LARC recurrence or metastasis was determined via imaging and/or histological analysis. Overall survival (OS) was defined as from the time of initial surgical resection to the date of death due to any cause. Without confirmation of death, recurrence, or metastasis, OS or FFR time was recorded based on the last known date that the patient was alive 14.

### Treatment and evaluation of tumor response

Radiotherapy was performed as previously described 15. Briefly, radiation was delivered via 6 and 10 MV photons using a three-field technique (posterior and bilateral) in most patients. Treatment was planned via computerized dosimetry, and a dose of 1.8 Gy per fraction was prescribed to cover the planning target volume. Radiotherapy was administered 5 days per week, once per day, at 1.8 Gy/d. Pelvic radiotherapy consisted of 45 Gy in 25 fractions over a period of 5 weeks, which was followed by a boost dose of 5.4 Gy administered in three fractions to the primary tumor. Preoperative chemotherapy was administered concurrently with radiation therapy. Concurrent chemotherapy was administered concurrently with radiation therapy and consisted of two cycles of capecitabine and leucovorin according to our institutional chemotherapy protocol 16. Capecitabine was administered orally at a dose of 1650 mg/m 2 per day, divided into two doses given 12 h apart. Leucovorin treatment (20 mg/m 2 per day) was also divided into two doses. Approximately 6 weeks after the completion of nCRT, the patients underwent definitive surgery. Pathologic evaluation of surgical specimens, including the primary tumor and resected nodes, was performed by a specialist pathologist. The complete absence of residual tumor cells in the primary tumor was designated pathologic complete response. Postoperative adjuvant chemotherapy was recommended in all patents except one who had operative wound disruption.

### Bioinformatic analysis

TIMER is a comprehensive resource for systematic analysis of immune infiltrates across diverse cancer types (https://cistrome.shinyapps.io/timer/) 17. TIMER applies a previously published statistical deconvolution method to infer the abundance of tumor-infiltrating immune cells (TIICs) from gene expression profiles 18. We analyzed the correlation of CXCL12, CXCR4, and FAPα expression with the abundance of immune cell infiltration.

Analysis of LARC datasets was carried out essentially as previously described 19. The raw data can be downloaded as GSE15781, GSE94104, and GSE133057. The CXCL12, CXCR4, and FAPα expression patterns were derived from GSE15781 and GSE94104 in order to compare the pre-nCRT LARC patient group with the post-nCRT LARC patient group. Pathway analysis was performed on the IBD datasets (GSE133057) using GSEA.

### Immunohistochemical Staining Analysis

Immunohistochemical staining of the FFPE tissue was conducted as previously described 20. Target Retrieval Solution, pH 9 (catalog #S2368, Dako, Glostrup, Denmark), was used for antigen revitalization. The tissue sections were incubated at 37 °C for 30 min with the mouse monoclonal anti-human CXCL12 (1:300, catalog #MA5-23759, Invitrogen, Rockford, IL, USA), the rabbit monoclonal anti-human CXCR4 (1:100, catalog #ab124824, Abcam, Cambridge, UK), and the rabbit polyclonal anti-human FAPα (1:20, catalog #AHP1322, BIO-RAD, Hercules, CA, USA).

In microscopic evaluation of the representative FFPE whole stain slides that included full-thickness sections of the tumor encompassing the deepest portion of the invasive front; the "invasive front of cancer cells after nCRT" was defined as the deepest point of invasion of cancer cells 21. The plasma membrane levels of CXCL12 and CXCR4 and cytoplasmic levels of FAPα in the deepest invasive front of cancer cells of LARC in the representative FFPE whole-tissue sample slides or high-grade dysplastic epithelial cells of adenoma were scored using the modified DAKO HercepTest TM Interpretation Manual—Breast Cancer Row version (where staining was scored as 0, 1, 2, or 3) (HercepTest™, Interpretation Manual Breast Cancer (available online: https://www.agilent.com/cs/library/usermanuals/public/28630_herceptest_interpretation_manual-breast_ihc_row.pdf) (accessed on 26 April 2020). The staining score of CXCL12 was as follows: 0, negative; 1, faint perceptible plasma membrane staining in less than 10% of the invasive front of tumor cells, visible at 200×; 2, perceptible plasma membrane staining in less than 10% of the invasive front of tumor cells, visible at 100×; and 3, plasma membrane staining in 10% and more than 10% of the invasive front of tumor cells, visible at 100×. Staining of 2 or 3 was regarded as positive expression of CXCL12. The staining score of CXCR4 was as follows: 0, negative; 1, only cytoplasmic expression; 2, perceptible plasma membrane staining in less than 10% of the invasive front of tumor cells; and 3, plasma membrane staining in 10% and more than 10% of the invasive front of tumor cells. Scores of 2 and 3 were regarded as positive expression of CXCR4. The staining score of FAPα was determined by cytoplasmic intensity as follows: 0, negative; 1, mild; 2, moderate; and 3, strong. Scores of 2 and 3 were regarded as positive expression of FAPα. The results were examined separately and scored by Kim, K-H, and Yeo, M-K, who were blinded to the patients' clinicopathological details. Any discrepancies in the scores were discussed to obtain a consensus.

### Statistical Analyses

Correlations of the clinicopathological parameters with expression of CXCL12, CXCR4, and FAPα were evaluated using Pearson's chi-square test. The differences in expression of the three proteins between adenoma, LARC after nCRT, and LARC untreated with nCRT were assessed using Pearson's chi-square test. Correlations between the three protein expression profiles were assessed using Spearman's correlation. Differences in paired pre-nCRT and post-nCRT protein expressions on each case were assessed using the Wilcoxon signed-rank test. Postoperative OS and FFR were determined using a log-rank test and univariate and multivariate Cox regression analyses. To compare the univariate differential expression in each dataset, we analyzed RNA data using the Wilcoxon-Mann-Whitney test with Benjamini-Hochberg FDR correction (*p* < 0.05, q < 0.25). Statistical significance was set at *p* < 0.05 (SPSS v.26; SPSS Inc., Chicago, IL, USA).

## Results

### CXCL12 expression is correlated with poor prognosis, immune cell infiltration, epithelial mesenchymal transition, and angiogenesis in LARC

**Figure 1:**
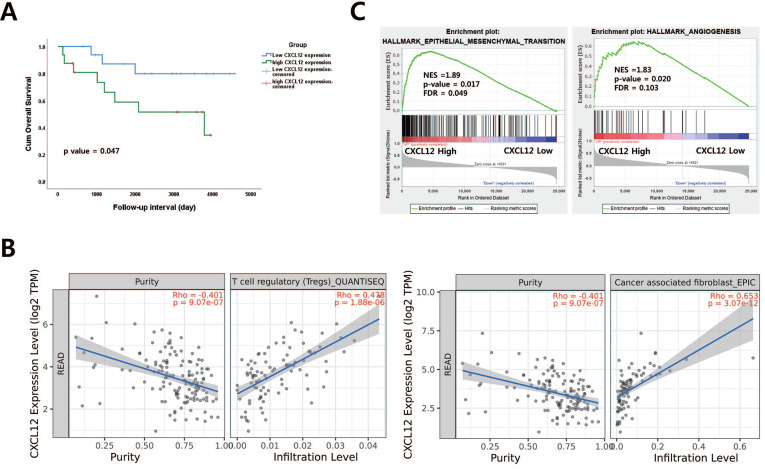
The relationship between CXCL12 mRNA expression and procancer pathways in locally advanced rectal cancer (LARC) (A) CXCL12 mRNA expression levels are significant prognostic factors for overall survival in GSE133057 (n=33, *p* = 0.047; log-rank test). (B) Correlation of CXCL12 expression with immune cell infiltration level in LARC. (C) Gene set enrichment analysis (GSEA) reveals correlation between CXCL12 expression and genes involved in epithelial-mesenchymal transition and angiogenesis. GSEA probing for enrichment of HALLMARK_EPITHELIAL_MESENCHYMAL_TRANSITION and HALLMARK_ANGIOGENESIS (READ, rectal adenocarcinoma).

To examine the correlation of CXCL12 mRNA in pretreatment LARC biopsy tissues with OS in LARC patients, a publicly available transcriptome dataset (GSE133057) was analyzed. The survival analysis revealed that those with high CXCL12 expression represented shorter overall survival than those with low CXCL12 expression (*p* = 0.047, Figure 1A). A previous study reported that FAPα (+) CAF is a principal source of CXCL12 and modulate the CXCL12-CXCR4 interaction to induce tumor immune evasion in PDAC and lung carcinoma-bearing mice 11, 22, 23. Therefore, the relationship between CXCL12/CXCR4 and FAPα was examined in patients with LARC using the TIMER database. A positive association was observed between CXCL12/CXCR4 and FAPα (cor = 0.589, *p*= 6.85 x 10 ^-17^, cor = 0.404, *p*= 6.54 x 10 ^-8^) (Supplementary Figure 1). However, CXCR4 and FAPα mRNA expression in pretreatment biopsy tissues were not correlated with OS in LARC patients (Supplementary Figure 2). To further corroborate the prognostic role of CXCL12, we identified a correlation between CXCL12 and immune cell infiltration of LARC using the TIMER database. Among various immune cells, T cell regulatory (Tregs), CAF, CD4+ T cells, macrophage, and dendritic cells were most strongly correlated with CXCL12 in LARC (Figure 1B and Supplementary Figure 3). Additionally, we performed gene set enrichment analysis (GSEA) using LARC datasets with CXCL12 gene signatures associated with more aggressive and invasive signatures including radioresistance and recurrence. We found the enrichment of epithelial-mesenchymal transition and angiogenesis signature was observed in groups with high CXCL12 expression (Figure 1C).

### Radiation-induced changes in CXCL12 and CXCR4 expression in LARC and adjacent normal tissues

**Figure 2:**
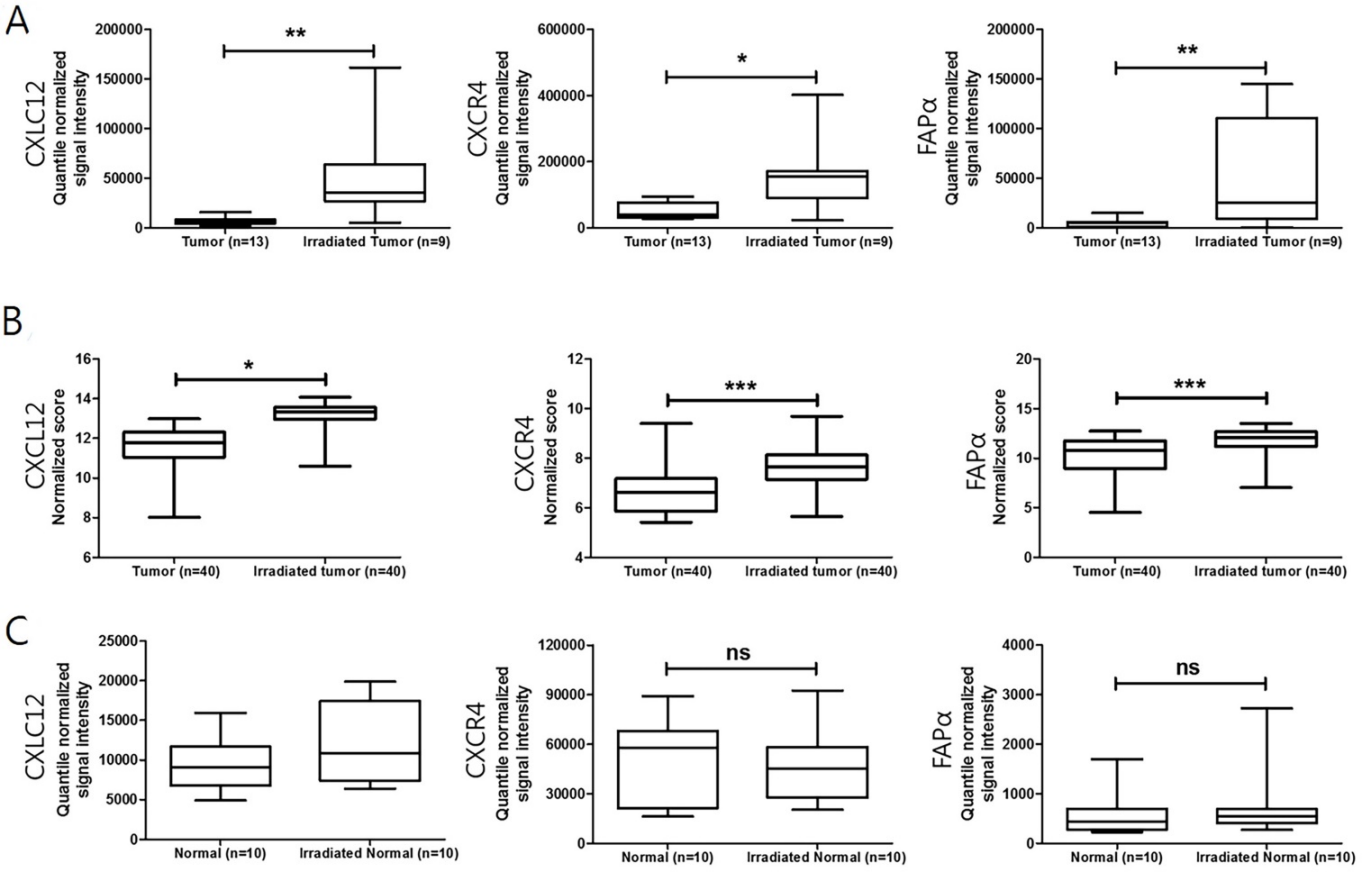
Changes in CXCL12, CXCR4, and FAPα expression after neoadjuvant chemoradiotherapy (nCRT) in LARC patients. (A) and (B) Boxplot of CXCL12, CXCR4, and FAPα expression in tumor and irradiated tumor tissues (using dataset GSE15781 and GSE94104). (C) Boxplot of CXCL12, CXCR4, and FAPα expression in normal and irradiated normal adjacent tissues (using dataset GSE15781). *p < 0.05, **p < 0.01, ns: not significant. Mann-Whitney U test (A, B, and C).

It was reported that radiation damages tumor vessels leading to tumor hypoxia and finally induces CXCL12 in patient-derived tumor xenografts 6. To evaluate the change in CXCL12, CXCR4, and FAPα expression between pretreatment and post-nCRT surgical specimens, a publicly available transcriptome dataset (GSE15781 and GSE94104) was analyzed (Figure 2). An increase in mRNA expression of CXCL12, CXCR4, and FAPα was observed in residual cancer tissues after nCRT treatment (Figure 2A and B). However, there was no significant difference in CXCL12, CXCR4, and FAPα expression when comparing pretreatment and post-treatment of normal tissues (Figure 2C). Changes in CXCL12 and CXCR4 levels in pre-nCRT biopsy and paired post-nCRT surgical specimen tissue samples from 45 LARC patients showed higher expression in LARC cancer cells after nCRT (*p* < 0.001 for each, Wilcoxon signed-rank test) (Supplementary Figure 4).

### Association of Clinicopathological Characteristics with Expressions of CXCL12, CXCR4, and FAPα

**Figure 3:**
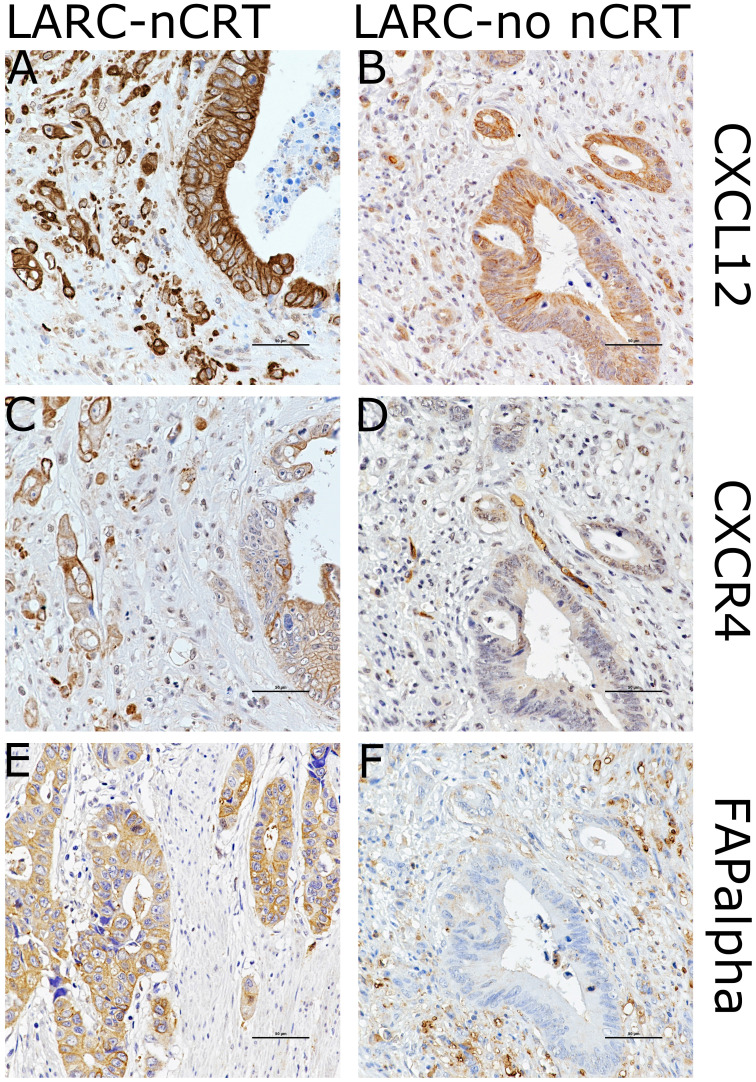
Images showing immunohistochemical staining of CXCL12, CXCR4, and FAPα in the case of each pathways in locally advanced rectal cancer (LARC) after neoadjuvant chemoradiotherapy (LARC-nCRT) (A,C,E) and LARC with no nCRT (LARC-no nCRT) (B,D,E). In the case of LARC-nCRT, the expression of CXCL12, CXCR4, and FAPα is higher than in the case of LARC-no nCRT (original magnification 400× ; scale bar= 50 µm).

The clinicopathological characteristics of the 103 LARC patients found to be associated with expression of CXCL12, CXCR4, and FAPα are presented in Table 1 and supplementary Table 1. The immunohistochemical staining of CXCL12 and CXCR4 exhibited a cytoplasmic and/or plasma membrane pattern. Staining categories of CXCL12 or CXCR4 were classified into positive or negative expression based on plasma membrane staining. FAPα immunostaining corresponded the most to a cytoplasmic pattern (Figure 3 and Supplementary Figure 5). Non-neoplastic epithelial cells and noninvasive carcinoma cells demonstrated little plasma membrane staining of either CXCL12 or CXCR4. The cytoplasmic levels of FAPα were significantly increased in invasive LARC cells after nCRT and tumor microenvironment immune cells in adenomas, while the adjacent non-neoplastic epithelial cells were negative.

**Figure 4:**
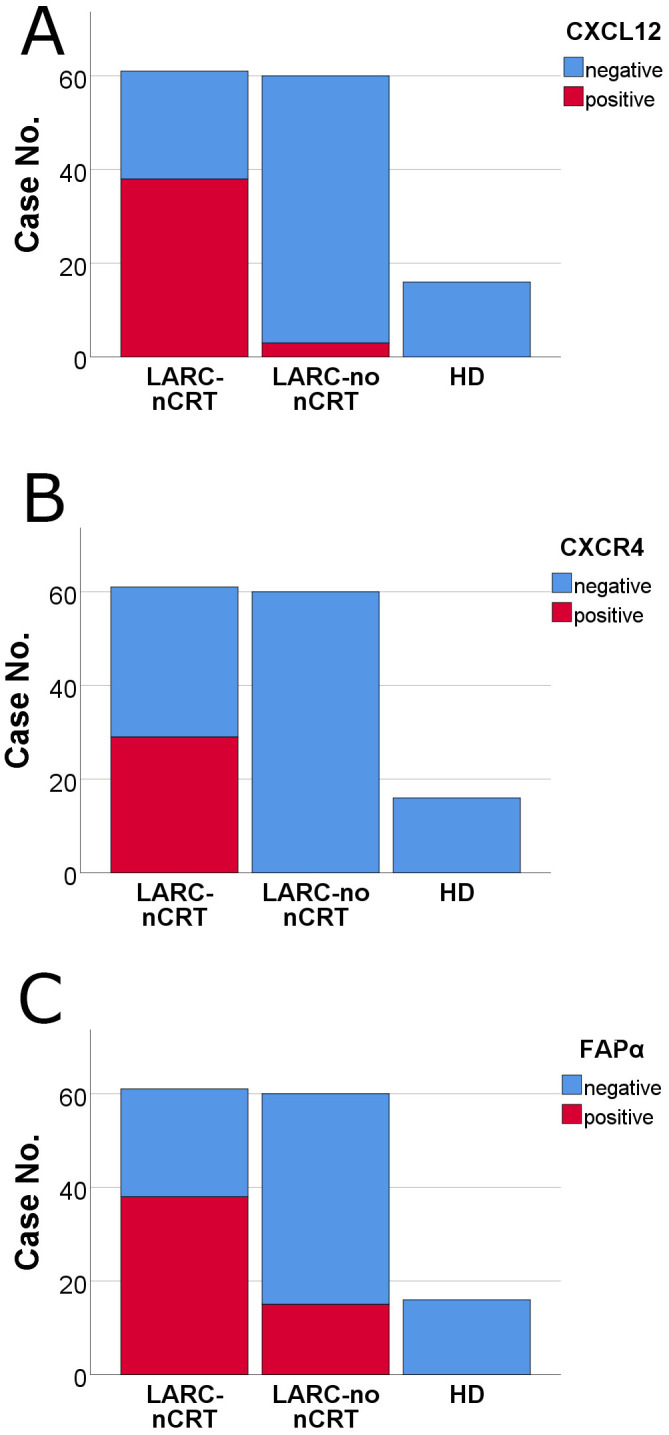
Comparison of the levels of CXCL12, CXCR4, and FAPα by immunohistochemical staining among pathways in locally advanced rectal cancer (LARC) after neoadjuvant chemoradiotherapy (nCRT) (LARC-nCRT) (n = 61), LARC untreated with nCRT (LARC-no nCRT) (n = 60) and high-grade dysplasia of colorectal adenoma (HD) (n = 16). The levels of the three proteins were higher in LARC-nCRT than in LARC-no nCRT or HD (p < 0.001 for each). (A) CXCL12 positivity: 62.3%, 38/61 LARC-nCRT cases; 5.0%, 3/60 LARC-no nCRT cases; and 0.0%, 0/16 HD cases. (B) CXCR4 positivity: 47.5%, 29/61 LARC-nCRT cases; 0.0%, 0/60 LARC-no nCRT cases; and 0.0%, 0/16 HD cases. (C) FAPα positivity: 62.3%, 38/61 LARC-nCRT cases; 25.0%, 15/60 LARC-no nCRT cases; and 0.0%, 0/16 HD cases.

CXCL12 expression in 121 cases of LARC was positively associated with lymph node metastasis and pTNM staging (*p* = 0.011 and *p* = 0.005, respectively). Levels of CXCL12 in the plasma membrane in 61 cases of LARC after nCRT showed a trend of positive expression in R score 2-3 compared to R score 0-1 (*p* = 0.084; Fisher's exact test). Furthermore, CXCL12, CXCR4, and FAPα levels showed more positive expression in LARCs after nCRT than in LARCs untreated with nCRT (*p* < 0.001 for each) (Figure 4). The staining of CXCL12, CXCR4, and FAPα was more intense in high-grade dysplastic epithelial cells and intramucosal invasive LARC cells than in the non-neoplastic epithelial cells of the 16 adenoma cases (*p* < 0.001 for each). There was a significant correlation of the CXCL12 levels in plasma membrane expression and CXCR4 and FAPα levels in the cancer cells of LARC after nCRT group (Spearman's Rho: *p* = 0.001 and *p* = 0.003) (Supplementary Table 2).

### CXCL12 levels are predictive of a shorter Disease-Free Survival period in 61 cases of locally advanced rectal cancer after neoadjuvant chemoradiotherapy

**Figure 5:**
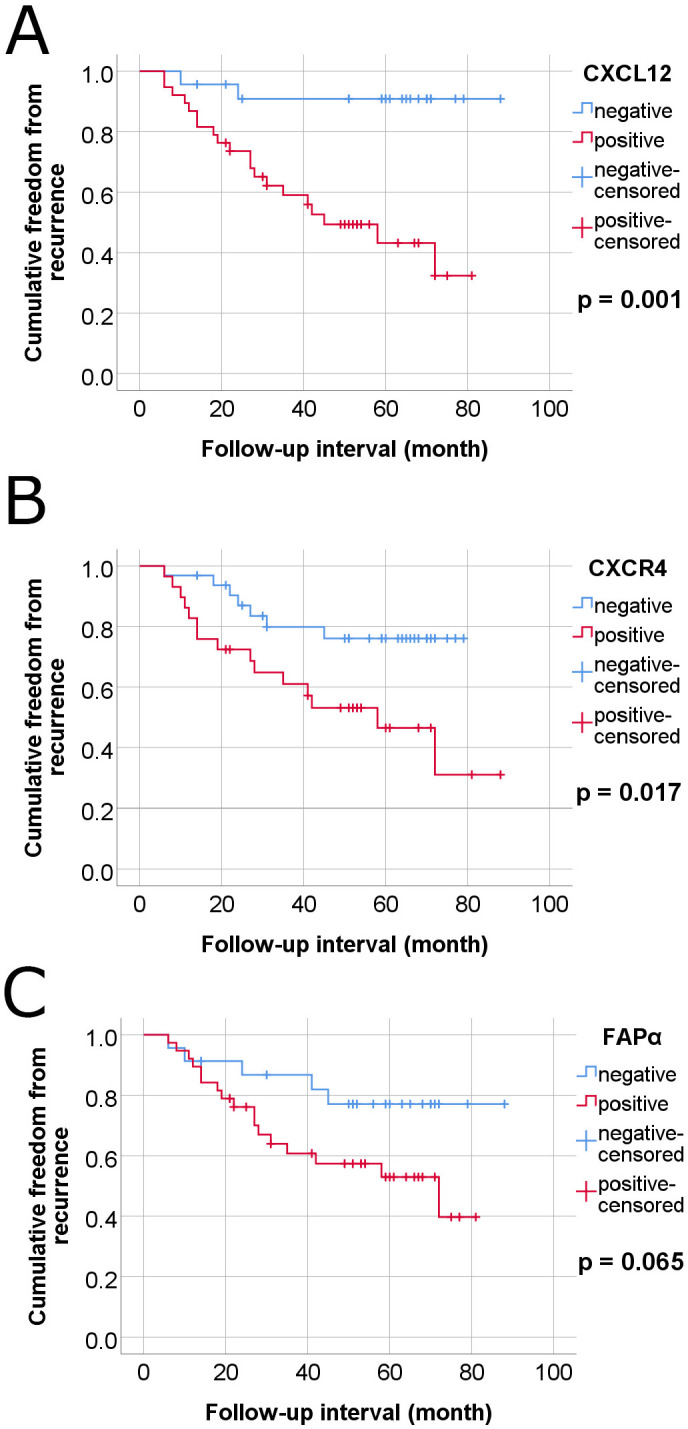
Kaplan-Meier survival curves-estermates of freedom from recurrence of colorectal cancer in 61 cases of locally advanced rectal cancer (LARC) with neoadjuvant chemoradiotherapy according to plasma membrane expressions of CXCL12, CXCR4 and FAPα in tumor cells. (A) CXCL12, (B) CXCR4, and (C) FAPα.

At a median follow-up of 45 months, 13/61 patients had died of nonspecified causes, 10 were alive with the disease, and 37 were alive with no evidence of the disease. Patients with positive CXCL12 expression in residual survival cancer cells displayed a positive association with worse FFR in univariate and multivariate Cox proportional hazard regression analysis (*p* = 0.005 and *p* = 0.031, respectively) (Table 2 and Table 3). On the other hand, CXCL12 expression in 60 cases of LARC untreated with nCRT did not obtain statistical significance in univariate or multivariate Cox regression analysis for OS and FFR (Supplementary Table 3, Supplementary Table 4). Kaplan-Meier curves also showed that positive CXCL12 expression was significantly associated with worse FFR (*p* = 0.001, long rank test) (Figure 5). Positive CXCR4 expression in plasma membrane showed an association with a shorter FFR period in univariate Cox proportional hazard regression analysis and long-rank test (*p* = 0.023 and *p* = 0.017, respectively) (Table 2, Figure 5).

## Discussion

In this study, bioinformatics approach was first used to investigate the role of CXLC12 in LARC using GSEA and TIMER database. The results revealed that CXCL12 expression is associated with poor prognosis, immune cell infiltration, epithelial mesenchymal transition, and angiogenesis in LARC. Then, the prognostic significance of CXCL12 expression was investigated in LARC using immunohistochemical study from our hospital. CXCL12 levels were elevated in the plasma membrane of LARC cancer cells after nCRT and positive expression of CXCL12 in the plasma membrane of LARC cells after nCRT was correlated with a shorter FFR period.

CXCL12 is a crucial chemokine in angiogenesis, apoptosis, proliferation, survival, differentiation, and metastasis, and is induced by hypoxia and growth arrest 24, 25. Hypoxia also induces the expression of the CXCL12 receptors CXCR4 and CXCR7, resulting in increased migration, adhesion, and survival of hypoxic cells 26, 27. Certain imbalances in the CXCL12/CXCR4/CXCR7 axis are associated with tumor immunosuppression and progression 11, 28. Therefore, it is becoming recognized emerging that the CXCL12/CXCR4/CXCR7 axis represents a potential target for cancer treatment. Some groups have reported that CXCR7 acts as a scavenger or decoy chemokine receptor for sequestering extracellular CXCL12 or modulating CXCR4 signaling 29-31. Previous studies have evaluated total protein or total mRNA expression of CXCL12 and its receptors CXCR4 and CXCR7 in stromal cells or colorectal cancer cells 32-34. Some studies have demonstrated reverse expression between CXCR4 and CXCL12 in colorectal cancer 35, 36, and CXCR4 expression correlated with poor prognostic survival outcomes 36. However, other reports have shown that CXCL12 expression in colorectal cancer cells was significantly correlated with poor prognostic factors including tumor budding grade and survival outcomes 34, 37. So far, little is known about the clinicopathologic significance of CXCL12 expression in the plasma membrane of cancer cells in patients with LARC treated with nCRT. We focused on the plasma membrane expression of CXCL12 in cancer cells in the invasive front region of LARC after nCRT.

Regarding the CXCL12/CXCR4 pathway, it was previously reported that concurrent radiotherapy and cisplatin chemotherapy increased CXCL12 mRNA expression and levels of phosphorylated CXCR4 (pCXCR4), pAKT, pERK, and PD-L1 in tumor cell regions, in addition to Ly6G and F4/80 in the stroma of the orthotopic uterine cervical cancer xenografts 38. Additionally, CXCR4 expression predicts recurrence-free and cancer-related survival in nCRT-treated LARC patients 36. Moreover, the addition of the CXCR4 inhibitor AMD3100 (plerixafor) along with radiotherapy and chemotherapy returned CXCL12, pCXCR4, pERK, PD-L1, Ly6G, and F4/80 expression to near control levels and was associated with a decreased tumor volume 38. AMD3100 was originally designed as an anti-HIV agent and later identified as an inhibitor of CXCR4, the co-receptor of T4- lymphotropic HIV strains 39. It was capable of mobilizing hematopoietic stem cells with the CD34 marker, so it has been approved by the US Food and Drug Administration for autologous transplantation of bone marrow cells in patients with non-Hodgkin's lymphoma or multiple myeloma 39-41. The CXCR4/CXCL12 axis plays an important role in local invasiveness and distant metastasis in a variety of cancers 39, 42. It is expected that various antagonists of CXCR4 could also be used to impair the development of cancer cell migration. Blocking vascular endothelial growth factor (VEGF) has been reported to lead to tumor hypoxia in local rectal cancer, which may lead to an increase in circulating CXCL12 associated with metastasis 43. Olaptesed pegol (NOX-A12) is an RNA oligonucleotide in L-configuration (Spiegelmer) that neutralizes CXCL12, and was developed for blocking CXCL12 in the tumor microenvironment 44, 45. In glioblastoma cells, the use of olaptesed pegol as a combination therapy has been reported to be effective in reducing the recruitment of tumor-associated macrophages due to CXCL12 increase after anti-VEGF therapy 46. A Phase II study, administering a four-week continuous infusion of plerixafor at the end of irradiation improves local control of glioblastoma 47. CXCL12 antagonists in combination therapy may be possible candidates for inhibiting metastasis in LARC after nCRT.

Classically, irradiation promotes a host immune response by exposing tumor-specific antigens that make tumor cells release a variety of substances promoting the priming and activation of cytotoxic T cells 48. However, a radiation-induced immunosuppressive phenomenon is suggested in light of the contradictory findings of several studies 49. Among these, there is growing interest in the role of CXCL12, CXCR4, and FAPα expression in cancer because of their immunosuppressive and procancer properties 50-52. Therefore, the correlation between CXCR4/CXCL12 expression and cancer treated with CRT has been studied by many studies. Retrospective studies showed that the persistant CXCR4/CXCL12 expression after CRT is associated with tumor aggressiveness and poor prognosis in LARC, esophageal cancer, cervical cancer and glioblastoma 32, 53-55. However, the exact mechanism how CXCL12 interact with immunosuppressive signals has yet to fully clarified. One of the potential mechanism is that CXCL12 may prevent tumor infiltrating lymphocytes from migrating close enough to lead to cell death 56. Furthermore, the expression levels of CXCL12 is associated with immunosuppressive cell infiltrations 52, 56. Similarly, our results showed that CXCL12 expression is strongest positive correlation with Treg and CAF infiltration mediating immunosuppressive microenvironment. These results imply that CXCL12 may serve as an important role in immune suppression in the irradiated tumor microenvironment.

It is thought that chemo- and radioresistant rectal cancer cells may be responsible for cancer recurrence. These cells are expected to have the characteristics of cancer stem cells, and CXCL12 expression has been reported to be related to this resistance. In previous studies, proliferation of the HT-29 cell line, a human colorectal adenocarcinoma cell line, was reduced by 5-FU or oxaliplastin treatment, but CXCL12-rich medium by coculture with a follicular dendritic cell line HK prevented the inhibitory effects of the chemotherapy drugs 57. Spheroid HT-29 cells were more resistant to 5-FU than that of adherent HT-29 cells. The spheroid HT-29 cells showed CD44 positivity of colorectal cancer stem cell feature, and had increased migration ability in the presence of CXCL12 58. Hypoxia enhanced the chemotactic activity of the human colon cancer cell line LoVo to CXCL12 59. FAPα, a type II membrane protein belonging to the serine protease family, has been known to be is up-regulated in activated fibroblasts of healing wounds, fetal mesenchymal tissues, and the tumor stroma of epithelial cancers 60, 61. FAPα, as a marker for CAFs, has been known to play a role in cancer growth, invasion, and metastasis by degrading the tumor microenvironment extracellular matrix and inducing epithelial-mesenchymal transition 61-64. FAPα is generally expressed in CAFs, but not in the actual cancer cells 65, 66. However, several studies have demonstrated that FAPα is also expressed in certain cancer cells, including colorectal cancer, pancreas cancer, stomach cancer and breast cancer 67-70. In previous studies, FAPα staining patterns have been described in colorectal cancer and pancreatic cancer 67, 68. In the first pattern, FAPα was mainly stained in carcinoma cells and staining in TIICs was insignificant. The second pattern was expressed opposite to the first pattern, exclusively restricted to TIICs. The third pattern was stained in both carcinoma cells and TIICs. In our results, the LARCs after nCRT showed the first pattern and the adenomas were the second pattern. The third pattern was seen in the LARCs untreated with nCRT. The mechanism for these patterns of FAPα expression has not yet been elucidated, but it is expected to be associated with tumor invasion and lymph node metastasis in the presence of complex epithelial-stromal cross-talk at the invasive front of cancer 67.

Radioresistance remains to be a major obstacle in the management of cancer patients. Although various studies investigated to identify novel and better radiosensitizers for the cancer treatment, the result still cannot reach clinical translation 71. Our results revealed that nCRT-induced mRNA expression of CXCL12, CXCR4, and FAPα in LARC. Interestingly, no changes in the mRNA expression of CXCL12, CXCR4, and FAPα in normal adjacent tissues were detected after nCRT. These results imply the cell-specific modulation of mRNA expression and suggest the involvement of different molecular mechanisms in cellular responses to ionizing in different cells. Advances in the knowledge of differential cellular responses to interventional chemoradiotherapy will provide opportunities for the development of new approaches that selectively enhance chemoradiotherapy in LARC.

In conclusion, this study indicates that nCRT elevates the levels of CXCL12 in the plasma membrane of LARC cells and is associated with cancer recurrence. LARC cells after nCRT demonstrated increased CXCL12 expression in the plasma membrane, suggesting a connection with their capacity for chemoradioresistance and to reinitiate tumor growth and metastasis. Inhibition of CXCL12 expression in the plasma membrane (CXCL12/CXCR4 signaling pathway) in LARC cells with combination therapies could be a potential strategy to improve the prognosis of LARC.

## Supplementary Material

Supplementary figures and tables.

## Figures and Tables

**Table 1:** Correlation of CXC chemokine ligand 12 (CXCL12), CXC chemokine type 4 (CXCR4), and fibroblast activation protein-α (FAPα) expression with clinicopathological factors in 121 patients with locally advanced rectal cancer.

Variable	No.	CXCL12			CXCR4			FAPα		
		(-)	(+)	*p* *	(-)	(+)	*p* *	(-)	(+)	*p* *
Gender		*N* = 80	*N* = 41	0.926	*N* = 92	*N* = 29	0.023	*N* = 68	*N* = 53	0.816
Male	79	52	27		55	24		45	34	
Female	42	28	14		37	5		23	19	
Age (years)				0.001			0.103			0.005
≤65	74	57	17		60	14		49	25	
>65	47	23	24		32	15		19	28	
nCRT				<0.001			<0.001			<0.001
No	60	57	3		60	0		45	15	
Yes	61	23	38		32	29		23	38	
(y)pT				0.663			0.277			0.213
2	44	28	16		31	13		28	16	
3	77	52	25		61	16		40	37	
(y)pN				0.011			0.386			0.067
0	75	56	19		59	16		47	28	
1-2	46	24	22		33	13		21	25	
(y)pTNM Stage				0.005			0.232			0.042
II	74	56	18		59	15		47	27	
III	47	24	23		33	14		21	26	

* Pearson's chi-square test; nCRT, neoadjuvant chemoradiotherapy; (y)pTNM stage, (neoadjuvant)pathologic stage in American Joint Committee on Cancer (AJCC) cancer staging system (8^th^ edition).

**Table 2:** Univariate analysis of overall survival and freedom from recurrence in 61 cases of locally advanced rectal cancer with neoadjuvant chemoradiotherapy.

Prognostic factor	Overall survival		Freedom from recurrence	
	HR (95% CI)	p*	HR (95% CI)	p*
**CXCL12 expression**		0.169		**0.005**
negative (n=23)	1 (reference)		1 (reference)	
positive (n=38)	2.494 (0.678-9.166)		7.960 (1.848-34.289)	
**CXCR4 expression**		0.196		**0.023**
negative (n=32)	1 (reference)		1 (reference)	
positive (n=29)	2.095 (0.683-6.432)		2.836 (1.154-6.968)	
**FAPα expression**		0.888		0.075
negative (n=23)	1 (reference)		1 (reference)	
positive (n=38)	1.083 (0.354-3.316)		2.477 (0.911-6.732)	
**Age at operation**		0.291		0.489
≤65 (n=31)	1 (reference)		1 (reference)	
>65 (n=30)	1.826 (0.597-5.589)		1.346 (0.580-3.128)	
**Sex**		0.373		0.490
Male (n=42)	1 (reference)		1 (reference)	
Female (n=19)	0.556 (0.153-2.025)		0.718 (0.281-1.838)	
**R score**		0.749		0.209
1 (n=11)	1 (reference)		1 (reference)	
2-3 (n=50)	0.809 (0.221-2.961)		2.557 (0.592-11.048)	
**ypTNM stage**		0.121		**0.021**
II (n=36)	1 (reference)		1 (reference)	
III (n=25)	2.419 (0.791-7.396)		2.783 (1.164-6.657)	
**Post-Chemotherapy**		0.527		**0.013**
No (n=14)	1 (reference)		1 (reference)	
Yes (n=47)	1.628 (0.360-7.349)		0.330 (0.137-0.794)	

* Univariate Cox regression analysis; HR, hazard ratio; CI, confidence interval; CXCL12 expression, plasma membrane expression in cancer cells; CXCR4 expression, plasma membrane expression in cancer cells; FAPα expression, cytoplasmic expression in cancer cells; R score, modified Ryan scheme for tumor regression score in American Joint Committee on Cancer (AJCC) cancer staging system (8^th^ edition); ypTNM stage, neoadjuvant pathologic stage in American Joint Committee on Cancer (AJCC) cancer staging system (8^th^ edition); and post-chemotherapy, post-operative adjuvant chemotherapy

**Table 3:** Multivariate analysis of overall survival and Freedom from recurrence in 61 cases of locally advanced rectal cancer with neoadjuvant chemoradiotherapy.

Prognostic factor	Overall survival		Freedom from recurrence	
	HR (95% CI)	P*	HR (95% CI)	P*
**CXCL12 expression**		0.363		**0.031**
negative (n=23)	1 (reference)		1 (reference)	
positive (n=38)	2.231 (0.396-12.577)		6.623 (1.183-37.078)	
**CXCR4 expression**		0.084		0.860
negative (n=32)	1 (reference)		1 (reference)	
positive (n=29)	3.822 (0.835-17.484)		1.120 (0.319-3.938)	
**FAPα expression**		0.284		0.868
negative (n=23)	1 (reference)		1 (reference)	
positive (n=38)	0.442 (0.099-1.968)		1.116 (0.305-4.080)	
**Age at operation**		0.098		0.182
≤65 (n=31)	1 (reference)		1 (reference)	
>65 (n=30)	3.146 (0.808-12.255)		0.465 (0.151-1.430)	
**Sex**		0.775		0.426
Male (n=42)	1 (reference)		1 (reference)	
Female (n=19)	0.804 (0.180-3.587)		0.642 (0.216-1.911)	
**R score**		0.109		0.829
1 (n=11)	1 (reference)		1 (reference)	
2-3 (n=50)	0.259 (0.050-1.349)		0.832 (0.157-4.415)	
**ypTNM stage**		0.191		0.222
II (n=36)	1 (reference)		1 (reference)	
III (n=25)	2.685 (0.611-11.803)		1.867 (0.685-5.087)	
**Post-Chemotherapy**		0.228		0.057
No (n=14)	1 (reference)		1 (reference)	
Yes (n=47)	2.858 (0.518-15.759)		0.307 (0.091-1.037)	

* Multivariate Cox regression analysis; HR, hazard ratio; CI, confidence interval; and R score, modified Ryan scheme for tumor regression score in AJCC cancer staging system (8^th^ edition); ypTNM stage, neoadjuvant pathologic stage in American Joint Committee on Cancer (AJCC) cancer staging system (8^th^ edition); and post-chemotherapy, post-operative adjuvant chemotherapy.

## Abbreviations

LARC: locally advanced rectal cancer
nCRT: neoadjuvant chemoradiotherapy
TNM: Tumor-Node-Metastasis
CXCL12: CXC chemokine ligand 12
CXCR4: CXC chemokine receptor type 4
FAPα: fibroblast activation protein-α
CAF: carcinoma-associated fibroblast
TIMER: the Tumor IMmune Estimation Resource
FFPE: Formalin-fixed paraffin-embedded
pT: pathologic tumor stage
pTNM: pathologic tumor, node, and metastasis
R score: modified Ryan scheme for tumor regression score
AJCC: the American Joint Committee on Cancer
FFR: the period of freedom from recurrence
OS: overall survival
TIICs: tumor-infiltrating immune cells
pCXCR4: phosphorylated CXCR4
VEGF: vascular endothelial growth factor
TILs: tumor-infiltrating lymphocytes
TME: tumor microenvironment
