# Supplementary material for: Elevated CXCL12 in the plasma membrane of locally advanced rectal cancer after neoadjuvant chemoradiotherapy: a potential prognostic marker
Source: J Cancer. 2022 Jan 1;13(1):162–73. doi: 10.7150/jca.64082 (PMC8692683; doi:10.7150/jca.64082)

Supplementary Table 1. Clinicopathological factors in the locally advanced rectal cancer cells treated with neoadjuvant chemoradiotherapy group and untreated with neoadjuvant chemoradiotherapy group

**Variable**	**No.**	**LARC-nCRT**	**LARC-no nCRT**	***p* ***
**Gender**		**61**	**60**	**0.406**
Male	79	42	37	
Female	42	19	23	
**Age (years)**				**0.019**
≤65	74	31	43	
>65	47	30	17	
**(y)pT**				**0.010**
2	44	29	15	
3	77	32	45	
**(y)pN**				**0.762**
0	75	37	38	
1-2	46	24	22	
**(y)p TNM stage**				**0.626**
II	74	36	38	
III	47	25	22	
**Post-Chemotherapy**				**0.636**
No	30	14	16	
Yes	91	47	44	

* Pearson's chi-square test; nCRT, neoadjuvant chemoradiotherapy; pStage, pathologic stage; LARC-nCRT, locally advanced rectal cancer cells treated with neoadjuvant chemoradiotherapy group; LARC-no nCRT, locally advanced rectal cancer cells untreated with neoadjuvant chemoradiotherapy group; ypTNM stage, neoadjuvant pathologic stage in American Joint Committee on Cancer (AJCC) cancer staging system (8th edition); and post-chemotherapy, post-operative adjuvant chemotherapy.

Supplementary Table 2. Correlations between the levels of CXCL12, CXCR4, and FAPα in the locally advanced rectal cancer cells treated with neoadjuvant chemoradiotherapy group using immunohistochemical staining.

Spearman's rho		CXCL12	CXCR4	FAPα
CXCL12	Correlation coefficient	1.000	0.402**	0.372**
	Sig. (2-tailed)	.	0.001	0.003
	No.	61	61	61
CXCR4	Correlation coefficient	0.402**	1.000	0.402**
	Sig. (2-tailed)	0.001	.	0.001
	No.	61	61	61
FAPα	Correlation coefficient	0.372**	0.402**	1.000
	Sig. (2-tailed)	0.003	0.001	.
	No.	61	61	61

**, $p < 0.01$

Supplementary **Table 3.** Univariate analysis of overall survival and freedom from recurrence in 60 patients with locally advanced rectal cancers untreated with neoadjuvant chemoradiotherapy.

Prognostic factor	Overall survival		Freedom From Recurrence	
	HR (95% CI)	*p**	HR (95% CI)	*p**
**CXCL12 expression**	0.521 (0.210-1.292)	0.160	1.069 (0.408-2.797)	0.892
**CXCR4 expression**	N/A	N/A	N/A	N/A
**FAPα expression**	0.859 (0.556-1.327)	0.493	1.301 (0.782-2.164)	0.311
**Age at operation**		0.007		0.963
≤65 (n=43)	1 (reference)		1 (reference)	
>65 (n=17)	3.286 (1.391-7.764)		1.032 (0.271-3.935)	
**Sex**		0.326		0.597
Male (n=37)	1 (reference)		1 (reference)	
Female (n=23)	1.537 (0.652-3.621)		1.380 (0.418-4.558)	
**pTNM stage**		0.424		0.224
II (n=38)	1 (reference)		1 (reference)	
III (n=22)	0.679 (0.264-1.752)		2.090 (0.638-6.850)	
**Post-Chemotherapy**		0.207		0.525
No (n=16)	1 (reference)		1 (reference)	
Yes (n=44)	0.567 (0.235-1.370)		1.645 (0.355-7.621)	
**Post-radiotherapy**		0.555		0.716
No (n=25)	1 (reference)		1 (reference)	
Yes (n=35)	0.773 (0.328-1.820)		1.256 (0.368-4.294)	

* Univariate Cox regression analysis; HR, hazard ratio; CI, confidence interval; CXCL12 expression, plasma membrane expression in cancer cells, scored as 0,1,2, or 3; CXCR4 expression, plasma membrane expression in cancer cells, scored as 0,1,2, or 3, All 60 cases are score 1.; FAPα expression, cytoplasmic expression in cancer cells, scored as 0,1,2, or 3; pTNM stage, pathologic stage in American Joint Committee on Cancer (AJCC) cancer staging system (8th edition); post-chemotherapy, post-operative adjuvant chemotherapy; and post-radiotherapy, post-operative adjuvant radiotherapy; and N/A, not applicable.

Supplementary **Table 4.** Multivariate analysis of overall survival and freedom from recurrence in in 60 patients with locally advanced rectal cancers untreated with neoadjuvant chemoradiotherapy.

Prognostic factor	Overall survival		Freedom From Recurrence	
	HR (95% CI)	*p**	HR (95% CI)	*p**
**CXCL12 expression**	0.499 (0.184-1.351)	0.171	0.764 (0.271-2.152)	0.610
**FAPα expression**	0.837 (0.541-1.295)	0.424	1.267 (0.738-2.174)	0.391
**Age at operation**		0.012		0.881
≤65 (n=43)	1 (reference)		1 (reference)	
>65 (n=17)	3.518 (1.313-9.424)		1.123 (0.246-5.117)	
**Sex**		0.508		0.734
Male (n=37)	1 (reference)		1 (reference)	
Female (n=23)	1.373 (0.537-3.505)		1.253 (0.341-4.600)	
**pTNM stage**		0.865		0.350
II (n=38)	1 (reference)		1 (reference)	
III (n=22)	0.909 (0.303-2.729)		1.990 (0.470-8.422)	
**Post-Chemotherapy**		0.928		0.785
No (n=16)	1 (reference)		1 (reference)	
Yes (n=44)	1.084 (0.190-6.201)		1.385 (0.133-14.461)	
**Post-radiotherapy**		0.803		0.883
No (n=25)	1 (reference)		1 (reference)	
Yes (n=35)	1.218 (0.259-5.722)		0.883 (0.168-4.641)	

* Multivariate Cox regression analysis; HR, hazard ratio; CI, confidence interval; CXCL12 expression, plasma membrane expression in cancer cells, scored as 0,1,2, or 3; FAPα expression, cytoplasmic expression in cancer cells, scored as 0,1,2, or 3; pTNM stage, pathologic stage in American Joint Committee on Cancer (AJCC) cancer staging system (8th edition); post-chemotherapy, post-operative adjuvant chemotherapy; and post-radiotherapy, post-operative adjuvant radiotherapy.

Supplementary Figure 1. Correlation between CXCL12/CXCR4 expression and FAPα in locally advanced rectal cancer (LARC). A significant positive correlation between CXCL12/CXCR4 and FAPα expression was observed in LARC.

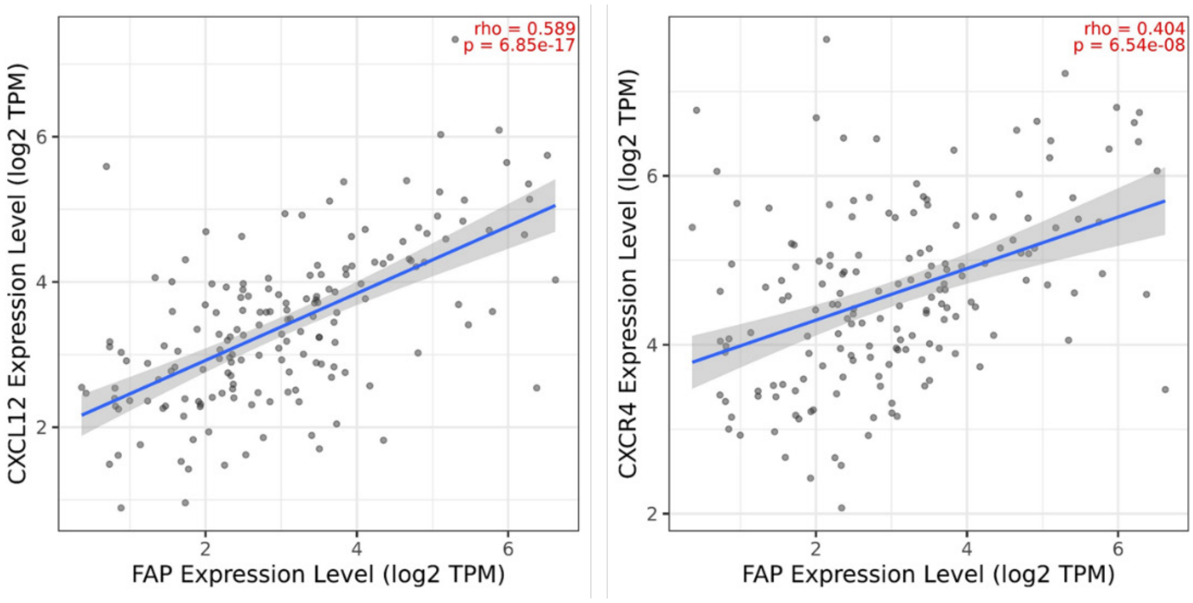

Supplementary Figure 2. Correlation between CXCR4 and FAPα expression and patient survival in locally advanced rectal cancer. Kaplan-Meier curves for low and high groups of the CXCR4 and FAPα gene expression in GSE133057 dataset. CXCR4 and FAPα mRNA expression levels are not significant prognostic factors for overall survival in GSE133057.

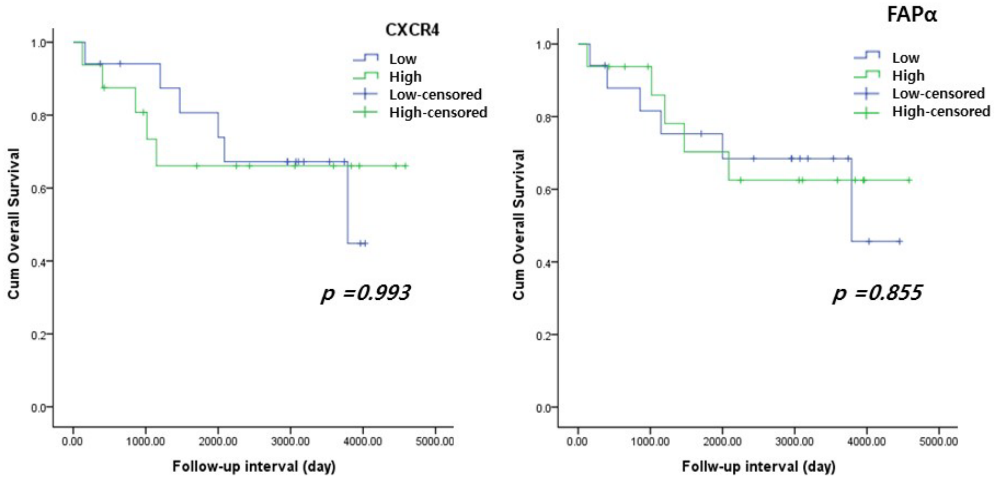

Supplementary Figure 3. Correlation of CXCL12 expression with immune infiltration level in LARC.

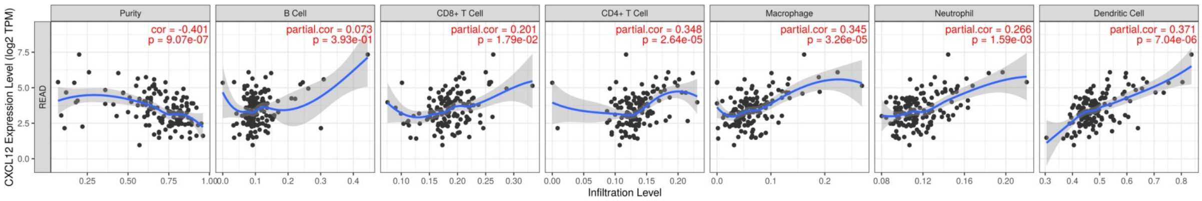

Supplementary Figure 4. Changes in CXCL12 and CXCR4 levels in pre-neoadjuvant chemoradiotherapy (nCRT) biopsy and paired post-nCRT surgical specimen tissue samples from 45 LARC patients showed higher expression in LARC cancer cells after nCRT ($p < 0.001$ for each, Wilcoxon signed-rank test).

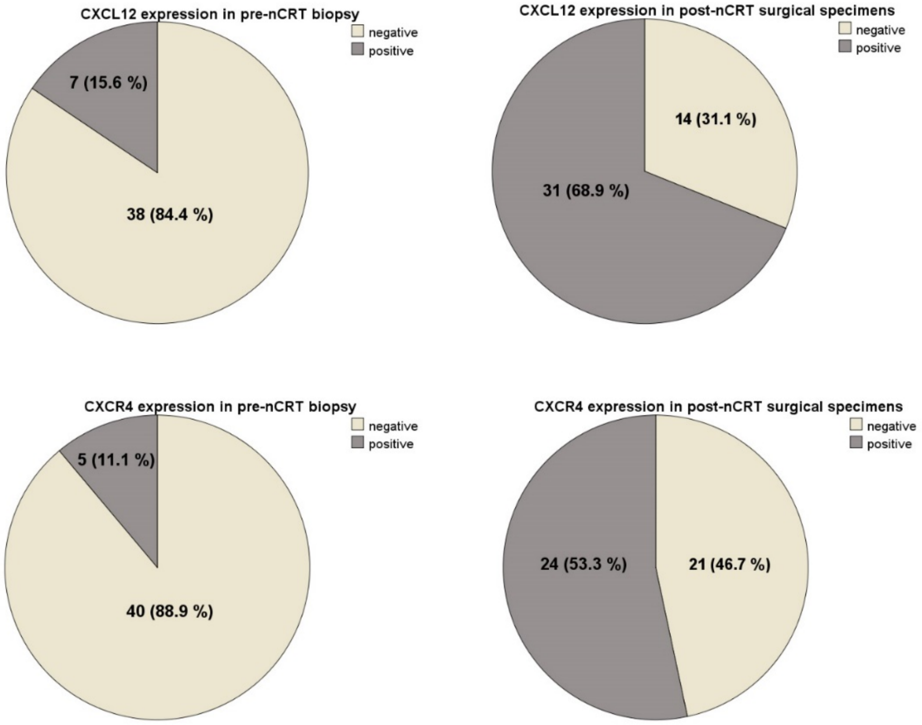

Supplementary Figure 5. Images showing immunohistochemical staining of CXCL12, CXCR4, and FAPα in the case of each pathways in non-neoplastic rectal glands (A, C, E) and rectal tubular adenoma (B, D, F). In both, CXCL12 and CXCR4 showed no plasma membrane expression (A, B, C, D) and FAPα exhibited little or no expression in the epithelial cells (E, F) (original magnification 400×; scale bar= 50 μm).

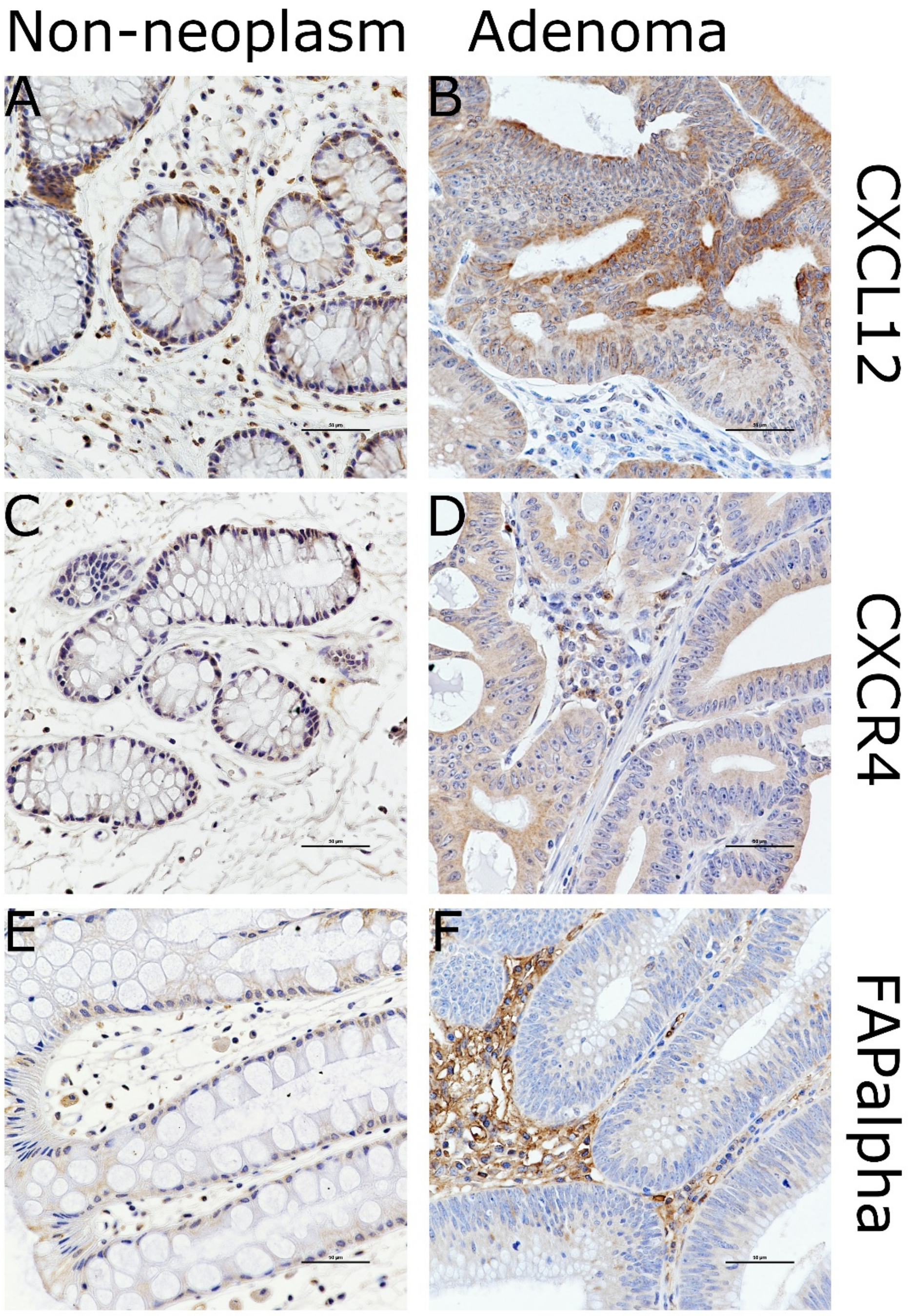

Supplement: Supplementary file 1 — Supplementary figures and tables. [file jcav13p0162s1.pdf]
